# Racial Differences in Exposure to Environmental Tobacco Smoke among Children

**DOI:** 10.1289/ehp.7379

**Published:** 2004-12-09

**Authors:** Stephen E. Wilson, Robert S. Kahn, Jane Khoury, Bruce P. Lanphear

**Affiliations:** ^1^Division of General Internal Medicine, University of Cincinnati, and; ^2^Division of General and Community Pediatrics, Cincinnati Children’s Hospital Medical Center, University of Cincinnati, Cincinnati, Ohio, USA

**Keywords:** African American, asthma, cotinine, ETS, housing

## Abstract

Exposure to environmental tobacco smoke (ETS) is a major cause of morbidity and mortality among U.S. children. Despite African-American children’s having a lower reported exposure to tobacco compared to whites, they suffer disproportionately from tobacco-related illnesses and have higher levels of serum cotinine than white children. The goal of this study was to test whether African-American children have higher levels of serum and hair cotinine, after accounting for ETS exposure and various housing characteristics. We investigated the level of cotinine in both hair and serum in a sample of 222 children with asthma. Using a previously validated survey for adult smokers, we assessed each child’s exposure to ETS. We collected detailed information on the primary residence, including home volume, ventilation, and overall home configuration. Despite a lower reported ETS exposure, African-American children had higher mean levels of serum cotinine (1.41 ng/mL vs. 0.97 ng/mL; *p* = 0.03) and hair cotinine (0.25 ng/mg vs. 0.07 ng/mg; *p* < 0.001) compared with white children. After adjusting for ETS exposure, housing size, and other demographic characteristics, serum and hair cotinine levels remained significantly higher in African-American children (β = 0.34, *p* = 0.03) than in white children (β = 1.06, *p* < 0.001). Housing volume was significantly associated with both serum and hair cotinine but did not fully explain the race difference. Our results demonstrate that, despite a lower reported exposure to ETS, African-American children with asthma had significantly higher levels of both serum and hair cotinine than did white children. Identifying causes and consequences of increased cotinine may help explain the striking differences in tobacco-related illnesses.

Environmental tobacco smoke (ETS) is a major cause of morbidity and mortality among children. ETS increases the risk of sudden infant death syndrome (SIDS), otitis media, lower respiratory tract infections, and asthma (Cook and Strachan 1999; Larsson et al. 2001). Furthermore, ETS contains known carcinogens, such as polycyclic aromatic hydrocarbons and 4-aminobiphenyl, which react with DNA and proteins to form adducts (Sexton et al. 2004; Tang et al. 1999). These compounds have been associated with the development of cancer (Perera et al. 2002; Tang et al. 2001). Data from the National Health Interview Survey indicate that regular smoking occurs in 36% of homes in which children reside, an estimate that far exceeds the Healthy People 2010 goal of reducing the percentage of children exposed to ETS to ≤ 10% [Schuster et al. 2002; U.S. Department of Health and Human Services (DHHS) 2000].

There is a disparity between the reported level of tobacco use and tobacco-associated outcomes among African Americans. Despite lower levels of reported tobacco use and exposure than among whites, African-American adults and children experience significantly higher levels of tobacco-related morbidity and mortality [Centers for Disease Control and Prevention (CDC) 1998]. African-American smokers experience significantly higher rates of smoking-related cancers when compared with white smokers, even though they report smoking fewer cigarettes per day (Caraballo et al. 1998, 2004; CDC 1998). African-American children experience higher rates of low birth weight, SIDS, and asthma, even though their reported exposure to ETS is less than that of white children. Although this paradox is not completely understood, many investigators hypothesize that racial differences in the metabolism of tobacco toxins may explain these striking differences in tobacco-related morbidity and mortality (Ahijevych and Garrett 2004; Ahijevych et al. 2002; Benowitz et al. 1999, 2004; Clark et al. 1996a; Mannino et al. 2001b; Perez-Stable et al. 1998; Tang et al. 1999).

Surprisingly, studies show that, despite lower levels of reported tobacco use compared with white smokers, African-American smokers have higher levels of some biologic markers of tobacco exposure. Until recently, most studies have relied on self-report to assess tobacco exposure. Increasingly, studies are incorporating biomarkers to objectively assess tobacco exposure (Al-Delaimy et al. 2001; Caraballo et al. 1998, 2004; Hecht et al. 2001; Klein and Koren 1999; Knight et al. 1996; Mannino et al. 2002, 2003). The most widely used biomarker is cotinine, which is a relatively stable product of nicotine metabolism. In laboratory experiments that controlled for tobacco smoke exposure, African Americans had serum cotinine levels that were 32–45% higher than those of whites (Benowitz et al. 1999, 2002; Perez-Stable et al. 1998). In a nationally representative sample, African-American smokers had significantly higher serum cotinine levels compared with white smokers, even though they reported smoking fewer cigarettes (Caraballo et al. 1998). However, the data for children and ETS exposure, rather than actual tobacco use, are more limited. In one Canadian study, black children had higher levels of urine and hair cotinine than did white or East Indian children, despite a lower reported home ETS exposure (Knight et al. 1996). In contrast, Mannino et al. (2001a) found no significant racial differences in serum cotinine among ETS-exposed children. Few studies involving children have systematically examined how key factors such as housing size, housing ventilation, and out-of-home exposure might influence the relationship between race, reported ETS, and cotinine (Henschen et al. 1997). Smaller housing size, for example, could be more common among African-American children and thus concentrate their exposure to ETS and increase cotinine levels.

The goal of the present study was to test whether African-American children with asthma have higher serum and hair cotinine levels compared with white children with asthma, even after accounting for reported ETS exposure both inside and outside of the home as well as important housing characteristics such as home volume and home ventilation.

## Materials and Methods

### Study design and subjects.

Data for this study were drawn from the Cincinnati Asthma Prevention (CAP) study. The CAP study is an ongoing double-blind, placebo-controlled trial, designed to test the efficacy of reducing ETS exposure using carbon-permanganate-zeolite (CPZ) high-efficient particulate air (HEPA) cleaners among children with asthma. We used the baseline data of the CAP study for our analysis. We identified potential subjects by using medical records and billing information from a large tertiary care center and a regional managed care organization, yielding subjects from urban, suburban, and rural communities. After notifying the child’s health care provider, we contacted the family by mail to describe the study in detail. Families that were interested in participating were contacted by telephone to determine their eligibility, and invited to participate in the trial. Eligibility criteria included physician diagnosis of asthma, exposure to five or more cigarettes per day in or around the home, electricity in the home, and no plans to move within the next 12 months. We excluded subjects who had a coexisting chronic lung disease, congenital heart disease, neuromuscular disease, or mental retardation.

### ETS reported exposure measure.

We adapted a questionnaire previously validated for adult smokers to systematically assess reported ETS exposure both in and outside the home (Coghlin et al. 1989). The primary caregiver reported the number of cigarettes smoked per day in the home by each resident of the household as well as the number of cigarettes smoked by regular visitors to the home. Using these data, we calculated the average number of cigarettes smoked per day in or around the home. For the previous day, we asked the caregiver to recall the number of hours the child spent inside the home and to estimate the level of ETS exposure in locations outside of the home. We also asked the parents whether their child had been exposed to tobacco smoke in a motor vehicle, a restaurant, an after-school program, or another home. We were particularly interested in the level of ETS exposure that occurred while traveling in a motor vehicle, because the small volume could concentrate the child’s exposure to ETS, thus increasing cotinine levels. We also documented the season when the visit occurred.

### ETS: biologic measures.

We collected both hair and serum samples from all study participants and analyzed them for cotinine. Cotinine has a serum half-life of approximately 15–21 hr and reflects ETS exposure in the prior 3–4 days (Ahijevych et al. 2002; Benowitz et al. 2002; Perez-Stable et al. 1998). Details of the analytic technique are presented elsewhere (Bernert et al. 1997, 2000). Briefly, we analyzed serum samples for cotinine using high-performance liquid chromatography (HPLC) linked to atmospheric-pressure chemical ionization tandem mass spectrometry. All samples were pre-screened for cotinine using an enzyme-linked immunoassay and categorized as either high or low concentration of cotinine. Samples with a high concentration of cotinine were diluted according to the laboratory protocol. Trichloroacetic acid was added to each specimen followed by potassium hydroxide to neutralize this mixture. Cotinine was extracted using methylene chloride and subsequently injected into the HPLC column. The eluant was monitored by mass spectrometry.

We determined longer-term exposure to ETS using hair cotinine. Cotinine enters the hair shaft through the hair-bulb blood supply, thus reflecting the average concentration in blood over a longer period of time (Al-Delaimy 2002; Al-Delaimy et al. 2002). Approximately 10 shafts of hair were cut at the root from the occipital region of each child. These hair samples were washed and dried with a mild detergent. Cotinine was extracted from the hair using sodium hydroxide. This solution was neutralized using hydrochloric acid. Cotinine concentrations were determined using radioimmunoassay as described by Klein and colleagues (Eliopoulos et al. 1994; Klein and Koren 1999). In this study, serum cotinine and hair cotinine were moderately correlated (*r* = 0.54, *p* < 0.001).

### Housing characteristics.

We collected detailed information about the home environment. An environmental technician visited the home, measured the total volume using an electronic tape measure and documented the age and general condition of the residence. The technician collected detailed information on the type and condition of the floors in each room, type of heating system, presence of an air conditioner or fans, and the overall design of the unit. During the home visit, we measured the level of particulate matter < 5 μm in diameter (PM_5_) using a Greentek-321 particle monitor (Met One Instruments, Inc., Grants Pass, OR). Particulate matter is highly correlated with air nicotine and thus can be used as an indicator of ETS (Cains et al. 2004).

### Race and sociodemographic covariates.

Primary caregivers were given a list of seven racial categories (African American or black, white, Asian or Asian American, Asian Indian, Native American, Native Hawaiian/Pacific Islander, Middle Eastern) along with Hispanic/Latino ethnicity from which to select the categories that best described their child. Parents were instructed to select as many of the categories as they deemed appropriate. Because there were few subjects in other racial and ethnic categories, only those children reported to be African American or white were included in our analysis. Children who were described as both African American and white were categorized as mixed-race subjects. We performed a sensitivity analysis with mixed-race subjects included with African-American subjects and then with white subjects to determine whether there were any differences. Additional measured covariates included insurance status, household income, parental education, parental marital status, and maternal depressive symptoms. Maternal depressive symptoms were assessed with the Beck Depression Inventory (Beck et al. 1996). We used a cutoff score of > 17 to indicate moderate to severe levels of depressive symptoms.

### Statistical analysis.

Serum and hair cotinine levels were both highly skewed. Thus, we log-transformed these variables before conducting any analyses. We compared subject characteristics using *t*-tests for continuous variables and chi-square tests for categorical variables. All values for cotinine are presented as geometric means. Using bivariate analysis, we compared a number of environmental characteristics between the two racial groups. We examined these factors to determine their association with both serum and hair cotinine. To account for seasonal variation, we documented the date of each home visit and grouped them into a particular season. Seasons were assigned using the following definitions: winter (January–March), spring (April–June), summer (July–September), fall (October–December). We compared these means using analysis of variance. We created a series of linear regression models including those factors that were associated with race and cotinine at a *p*-value ≤ 0.25. The first model contained only the race variable (model 1). Subsequent models included both race and one or more covariates. From this series of models, we selected factors that changed the race estimate by at least 5–10% and these factors were included in the final model. Although age, sex, and season of the year did not meet our definition of confounding, we included them in our final analysis for face validity of our model. We tested for effect modification by introducing a race–ETS product term in our multivariable regression models. We assessed the residuals of the final models for normality. All analyses were completed in SAS version 8.2 (SAS Institute Inc., Cary, NC).

## Results

**Figure 1:**
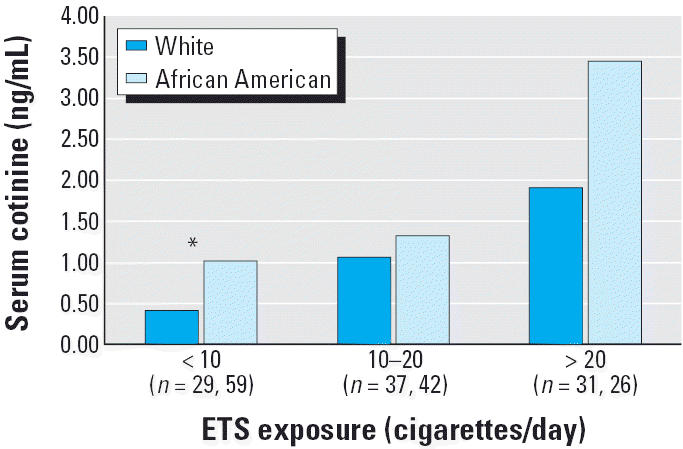
Geometric mean values of serum cotinine stratified by home ETS exposure (as measured by the reported number of cigarettes smoked per day in or around the home) for children with asthma stratified by race.
*p < 0.05.

**Figure 2:**
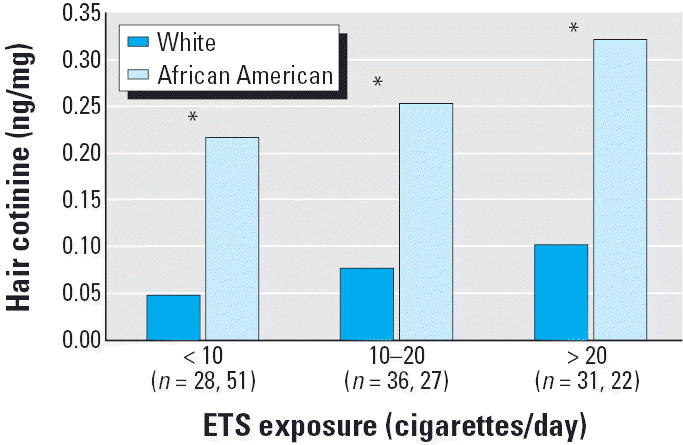
Geometric mean values for hair cotinine stratified by home ETS exposure (as measured by the reported number of cigarettes smoked per day in or around the home) for children with asthma stratified by race.
*p < 0.05.

Of the 222 children we selected for this study, 52% were described as African American, 45% were described as white, and 3% were described as African American and white. African-American children were slightly older than white children, but there were no significant differences in sex or parental education by race (Table 1). African-American children were more likely to reside in single-parent and low-income households and to have public insurance compared with white children. African-American children were reportedly exposed to fewer cigarettes per day in or around the home than white children. Despite this lower reported exposure to ETS, African-American children had significantly higher levels of both serum and hair cotinine (Table 2). When stratified by reported level of ETS exposure, African-American children had higher levels of serum and hair cotinine (Figures 1 and 2). Lower levels of PM_5_ in African-American homes offered some confirmation of their lower reported exposure to ETS. Among the factors that might influence ETS exposure, we found that African-American children had smaller home volumes, were less likely to have fans or an air conditioner, and were less likely to be exposed to ETS in a car.

## Discussion

Our results demonstrate that, despite lower reported exposure to ETS, African-American children have significantly higher levels of both serum and hair cotinine. These findings were partially explained by smaller home sizes among African-American children. Still, the racial differences in cotinine persisted after accounting for housing characteristics and exposures that occurred outside the home. Consistent with this study, Knight et al. (1996) similarly found that black children had significantly higher levels of cotinine compared with white children, despite lower ETS exposure. In contrast, Mannino et al. (2001a) found no differences by race in a cohort of tobacco-exposed children. However, neither study accounted for housing characteristics or ETS exposure outside the home. Our study clearly demonstrates that even after accounting for reported exposure and potential modifying environmental factors, African-American children have significantly higher levels of both serum and hair cotinine.

There are at least two possible explanations for why African Americans may have higher levels of cotinine. One explanation could be a racial difference in their metabolism of tobacco-related products. A number of recent studies have found that African-American smokers metabolize nicotine and cotinine more slowly than do white smokers. Perez-Stable and colleagues infused deuterium-labeled nicotine and cotinine into subjects and monitored for nicotine and cotinine clearances (Benowitz et al. 1999, 2004; Perez-Stable et al. 1998). They found that African-American subjects had a higher total clearance and nonrenal clearance of cotinine and longer serum half-life of cotinine. Some authors hypothesize that polymorphisms in the cytochrome P450 2A6 (*CYP2A6*) gene might explain racial differences in enzyme levels and enzyme activity (Ahijevych et al. 2002; Benowitz et al. 1999, 2004; Tricker 2003). However, the size of the cotinine differences observed in this study would likely require quite substantial genetic variation. Results from a study completed by Paschke et al. (2001) suggest that *CYP2A6* polymorphisms occur infrequently in both African-American and white populations, 0.3 and 1.3%, respectively. Small differences in the prevalence of *CYP2A6* polymorphisms may not fully explain the striking differences in cotinine and health outcomes. Thus, the attribution of a genetic basis for racial differences in cotinine should be made cautiously, because race is at best a crude marker for genetic variation (Cooper 2003; Cooper et al. 2003).

Differences in additives to cigarettes commonly smoked by African Americans could also explain the observed racial differences in cotinine. Approximately 80% of African-American smokers report using mentholated tobacco products, compared with 20% of white smokers (Kabat et al. 1991). Although a complete understanding of this preference is unclear, it is well documented that tobacco companies have targeted their marketing campaigns of mentholated brands toward African Americans (Balbach et al. 2003). On average, mentholated brands have significantly higher levels of tar and nicotine (Federal Trade Commission 2000, 2002). In a randomized crossover trial, Benowitz et al. (2004) found that African-American subjects had higher levels of both serum nicotine and blood carboxyhemoglobin while smoking mentholated tobacco products than when they were smoking nonmentholated tobacco products. Ahijevych et al. (2002) found that white and African-American women who smoked mentholated cigarettes had higher levels of serum cotinine compared than did white women who smoked nonmentholated tobacco products. Because no African-American women smoked nonmentholated products, this group could not be assessed. Although these studies demonstrate an effect of menthol on nicotine and cotinine metabolism, they were all completed in adult smokers. It is not clear whether this relationship between menthol and cotinine exists among children exposed to ETS.

Although racial differences in cotinine were present for hair and serum, the relative racial difference was greater for hair cotinine. The larger racial difference for hair cotinine compared with serum cotinine may be due to multiple factors. First, there may be less variability in hair cotinine. Serum cotinine measures short-term ETS exposure (3–4 days), whereas hair cotinine measures ETS exposure in the prior month (Al-Delaimy 2002; Al-Delaimy et al. 2002). Because hair cotinine measures long-term ETS exposure, it is less vulnerable to everyday variability in ETS exposure and metabolism (Al-Delaimy 2002). Second, there may be racial differences in the use of dyes and hair treatments, which could affect hair cotinine levels. Pichini et al. (1997) found that use of dyes and hair treatments decreased levels of hair cotinine. Last, there may be differences in other unmeasured factors. Despite these relative differences, our results consistently demonstrate that African-American children have higher levels of cotinine in both hair and serum.

Racial differences in cotinine metabolism raise questions about whether cotinine could help explain the racial differences in tobacco-related morbidity and mortality. Although cotinine is often considered an inert biomarker, recent literature suggests that cotinine exhibits biologic activity. Cotinine is mitogenic on vascular smooth muscle cells in a dose-related fashion (Carty et al. 1997) and has been shown to reduce the survival of hippocampal neurons and to decrease neurite function (Audesirk and Cabell 1999). Further research is necessary to ascertain whether prolonged exposure to nicotine and cotinine contributes to the observed racial differences in childhood asthma morbidity and mortality.

Our study has some limitations. First, we measured ETS exposure using parent report. A systematic underreporting of tobacco use by African-American parents could explain the racial differences in serum and hair cotinine. However, prior studies suggest this is unlikely (Caraballo et al. 2001, 2004; Clark et al. 1996b; Wills and Cleary 1997). For example, Clark et al. (1996b) found no racial difference in the reporting of tobacco use relative to the number of cigarette butts collected. Furthermore, our study measured the level of particulate matter in the main activity room at each residence. Consistent with lower reported ETS exposure, we found that African-American residences had significantly lower levels of particulate matter. Data from other tobacco studies indicate that particulate matter correlates well with ETS; indeed, ETS is the major contributor of indoor particulates (Cains et al. 2004; Wallace et al. 2003). Nevertheless, additional studies that objectively measure air nicotine are needed. Second, we did not collect information on cigarette type and thus cannot account for the potential effect of ETS exposure from mentholated cigarettes. As mentioned above, menthol has been associated with elevated levels of serum cotinine in active smokers. Finally, we grouped individuals into broad categories by race. Race is not a biologic construct, but an imprecise categorization that is a proxy for environmental, cultural, socioeconomic, and biologic differences. We need additional research to identify the specific factors that increase the risk of African-American individuals having higher levels of cotinine.

In summary, our study demonstrates that African-American children with asthma who were, on average, exposed to lower levels of ETS had higher levels of both serum and hair cotinine compared with white children with asthma. If African-American children are more susceptible to tobacco-induced toxicity, then we should target additional public policy initiatives toward reducing ETS exposure among this population. Further studies are needed to determine whether there are racial differences in the metabolism of other tobacco-related toxins and to assess the efficacy of interventions to reduce ETS exposure among all children.

## Figures and Tables

**Table 1:** Demographic characteristics of children in the CAP study, by race.

Characteristic	Total (*n* = 222)	White (*n* = 97)	African American (*n* = 125)	*p*-Value*^a^*
Age [years (mean ± SD)]	8.6 ± 1.8	8.4 ± 1.7	8.8 ± 1.7	0.046
Sex (%)				
Female	38.3	37.1	39.2	0.75
Parental education (%)				
Less than high school	18.5	19.6	17.6	0.67
High school graduate	46.9	42.3	50.4	
Some college	23.0	24.7	21.6	
College graduate	11.7	13.4	10.4	
Parental married status (%)				
Married	37.8	63.9	17.6	< 0.001
Divorced	11.7	18.6	6.4	
Single never married	41.9	11.3	65.6	
Separated/widowed	8.6	6.2	10.4	
Household income (%)				
< $20,000	41.4	27.8	52.0	< 0.001
$20,000–40,000	30.2	25.8	33.6	
> $40,000	24.3	41.2	11.2	
Missing	4.1	5.2	3.2	
Insurance status (%)				
Private insurance	42.8	67.0	24.0	< 0.001
Public insurance	51.4	27.8	69.6	
Uninsured	5.9	5.2	6.4	
Maternal depression (%)*^b^*	27.5	23.7	30.4	0.27
Season of visit (%)				
Winter	27.5	25.7	28.8	0.47
Spring	27.5	26.8	28.0	
Summer	28.8	26.8	30.4	
Fall	16.2	20.6	12.8	

a Comparison of African-American versus white children using chi-square or *t*-tests as appropriate.

b Depression determined using a Beck Depression Inventory score > 17.

**Table 2:** ETS exposure, cotinine, and housing characteristics by race.

Covariates	Total	White	African American	*p*–Value*^a^*
Home ETS exposure (cigarettes/day)	16.5 (14.9–18.1)	18.7 (16.3–21.0)	14.9 (12.8–14.9)	0.013
Serum cotinine (ng/mL)*^b^*	1.2 (1.01–1.42)	0.97 (0.74–1.27)	1.41 (1.14–1.75)	0.03
Hair cotinine (ng/mg)*^b^*	0.14 (0.12–0.17)	0.07 (0.06–0.09)	0.25 (0.20–0.31)	< 0.001
PM_5_ (μg/m^3^)*^b^*	3,791 (3,328–4,318)	4,634 (3,842–5,590)	3,237 (2,716–3,857)	0.007
Home volume (m^3^)*^c^*	228 (215–241)	249 (225–272)	212 (199–226)	0.01
Time at home (hr)*^c^*	16.8 (16.2–17.4)	16.0 (15.0–17.0)	17.3 (16.6–18.1)	0.04
Car ETS exposure (%)	25.7	34.0	19.2	< 0.001
Air conditioner (%)	71.6	82.4	63.2	0.0016
Fan in home (%)	36.0	46.4	28.0	0.005
Open floor plan (%)	5.0	3.1	6.4	0.26
Public ETS exposure (%)	3.6	5.2	2.4	0.27
Carpet in bedroom (%)	85.1	92.8	79.2	0.0048
Carpet in main room (%)	76.6	82.5	63.2	0.0016

a Comparison of African American versus white using chi-square or *t*-test as appropriate.

b Data are expressed as geometric mean (95% confidence interval).

c Data are expressed as arithmetic mean (95% confidence interval).

**Table 3:** Associations between cotinine and various covariates.

Covariates	Serum cotinine (ng/mL)	*p*-Value	Hair cotinine (ng/mg)	*p*-Value
Correlation coefficients				
Age	−0.039	0.56	0.31	0.66
Home volume (m^3^)*^a^*	−0.370	< 0.0001	−0.287	< 0.0001
Time at home (hr)*^a^*	−0.047	0.49	0.192	0.79
Geometric means for cotinine				
Car ETS exposure				
No	1.05	0.01	0.14	0.76
Yes	1.75		0.15	
Air conditioner				
No	2.25	0.01	0.21	0.01
Yes	1.04		0.15	
Fan in home				
No	1.31	0.18	0.15	0.21
Yes	1.03		0.12	
Home configuration				
Open	1.20	0.95	0.14	0.89
Closed	1.17		0.15	
Public ETS exposure				
No	1.19	0.70	0.14	0.44
Yes	1.40		0.20	
Carpeting in activity room				
No	1.25	0.80	0.21	0.0149
Yes	1.18		0.12	
Carpeting in bedroom				
No	1.77	0.06	0.24	0.013
Yes	1.12		0.13	
Maternal depression				
No	0.96	0.0001	0.12	0.0107
Yes	2.13		0.20	
Season of the year				
Winter	1.63	0.07	0.14	0.013
Spring	1.30		0.15	
Summer	0.93		0.19	
Autumn	0.99		0.08	

a Pearson correlation coefficients.

**Table 4:** Multivariable regression for the mean change in log serum cotinine (± SE).

Covariates	Model 1 (β)	Model 2 (β)	Model 3 (β)	Model 4 (β)	Model 5 (β*^a^*)
African American	0.38 ± 0.17	0.56 ± 0.15	0.62 ± 0.16	0.44 ± 0.15	0.34 ± 0.16
ETS exposure at home (cigarettes/day)		0.05 ± 0.01	0.04 ± 0.01	0.05 ± 0.01	0.04 ± 0.01
ETS exposure in the car			0.48 ± 0.18	0.41 ± 0.16	0.43 ± 0.17
Home volume (100 m^3^)				−0.45 ± 0.07	−0.42 ± 0.07

Model 5 adjusts for age, sex, fan, air conditioner, maternal depression, and season in addition to covariates listed above.

a Final model adjusted *r*^2^ = 0.35, model *p* < 0.0001.

**Table 5:** Multivariable regression for mean change in log hair cotinine (± SE).

Category	Model 1 (β)	Model 2 (β)	Model 3 (β)	Model 4*^a^* (β)
African American	1.22 ± 0.16	1.30 ± 0.16	1.21 ± 0.16	1.06 ± 0.17
ETS exposure at home (cigarettes/day)		0.02 ± 0.01	0.02 ± 0.01	0.02 ± 0.01
Home volume (100 m^3^)			−0.28 ± 0.07	−0.30 ± 0.08

Final model adjusted for age, sex, air conditioner, maternal depression, bedroom carpeting, main activity room carpeting, and season in addition to the covariates listed above.

a Final model adjusted *r*^2^ = 0.33, model *p* < 0.0001.
